# Bibliographical Notices

**Published:** 1846-07

**Authors:** 


### BIBLIOGRAPHICAL NOTICES.

*Fevers: Their Diagnosis, Pathology, and Treatment. Prepared and edited, with large additious, from the Essays on Fever in Tweedie's Library of Practical Medicine.* By Meredith Clymer, M. D., Pro essor of the Principles and Practice of Medicine in the Franklin Medical College of Philadelphia, etc. etc. 8vo. pp. 604. Lea & Blanchard; Philadelphia, 1846.

The essays on fever contained in Tweedie's Library of Practical Medicine, as is well known, are the productions of several different authors. These, collated with recent authorities, including essays and notices in the journals, together with frequent comments and additions, and a chapter of thirty odd pages on "*typhoid fever*" by Dr. Clymer, constitute the present publication. A work so made up, must necessarily be exceedingly heterogeneous in its character. No unity of general principles, whether of pathology or therapeutics, is to be expected. As each writer, however, treats the subject after his own fashion, the various essays afford materials for instructive enquiry and comparison, which, to thinking minds, may well compensate for the distraction occasioned by the unharmonious whole.

The various essays contained in Tweedie's Library, which, we have said, constitute the basis of this publication, have been long before the profession, and have been extensively noticed and commented on in the journals, and are therefore scarcely subjects for criticism at the present time; while the additions by the editor, under each head, although evincing much careful enquiry and judicious selection, being for the most part references to statements and opinions, often very discordant, equally forbid such an examination. On every subject discussed, much valuable information is given, which may be profitably studied by all whose duty it is to treat such complaints, and if space permitted, we should be tempted to extract numerous examples for the edification of our readers. One or two, however, must suffice.

The frequent prevalence of puerperal fever, of late years, in our lying-in hospitals and large cities, and the great mortality attending upon it, have rendered it a subject of peculiar interest to the American practitioner—an interest much enhanced by the ultra, exclusive and opposite views maintained by writers and teachers of the highest authority. On this account, we shall extract a very few remarks from the essay on that subject contained in this publication written by Dr. Locock, merely premising that Dr. L. is a gentlemen of great experience in his profession, whose high reputation has caused him to be selected as the physician-accoucheur of her Majesty, Queen Victoria.

"On perusing the numerous treatises that have been published within the last half century on this highly important class of diseases, the reader must necessarily be struck with the very extraordinary differences of opinion amongst the several writers as to the history and nature of the disease, the symptoms, mode of treatment, and the result of the practice adopted. The only point on which all seem to be agreed is, its great and striking fatality, and that it is one of the most serious, intractable, and destructive maladies to which puerperal women are liable.

"What is of practical importance in these different histories may be easily reconciled, without attributing to any of these authors erroneous statements or wilful perversions. They have each described what they saw, fairly and completely, and the same difference of opinion as to the nature of the disease exists to the present day. The confusion has arisen chiefly from considering that every form of fever to which puerperal women are liable is necessarily the same, the truth being that they vary in their nature and treatment as much as other kinds of fevers. With this precautionary consideration there can be no reasonable objection to the term '*puerperal fevers,*' which has so often been caviled at and attempted to be reformed. All the other names which have been substituted are liable to objection, as assuming some particular structure to be invariably attacked with disease, whereas the local affection is by no means uniform; and hence the term 'puerperal peritonitis,' 'peritoneal fever,' 'inflammation of the uterus and its appendages in puerperal women,' will be apt to mislead. The name '*puerperal fevers,*' compromises no opinion; it does not necessarily imply the existence of idiopathic puerperal fever, a doctrine now so much disputed; and whatever may be our own view of the subject, we beg distinctly to state, that in selecting this name we only wish to use one most easily understood and generally recognized.

"From our own researches into the writings of others, and from personal experience, both in private practice and as attached for nearly eighteen years to a very large lying-in hospital, we are inclined to doubt the propriety of considering puerperal fever as merely symptomatic of *local inflammation.* But if this were admitted, we have records of so great a variety in the seat of the inflammatory action, as proved upon dissection, that it will be found impossible to select any one part as peculiarly affected. We find in other fevers a liability to particular local lesions, equally varying; the brain, the chest, the mucous membrane of the stomach and bowels, have been, in different epidemics, the seats of the disorganizing process; but it has been too much the fashion to put down all the morbid appearances as exclusively the cause, and not the result, of the constitutional affection. In considering puerperal fevers it has long been our conviction, that what has been called by SYDENHAM *the constitution of the year,* has been too much lost sight of. The great difference in the accounts of puerperal fevers by different writers is thus easily explained:—They have seen and described epidemics differing in their type, their local accompaniments, and their power of being influenced by remedies; and hence, honestly stating exactly what they saw, we have an explanation of what would otherwise appear contradictory. That the fevers of puerperal women are much influenced by the character of the other fevers of the season, was strikingly exemplified in the Westminister Lying-in Hospital during the spring of 1838, when some of the fatal cases were attended by eruptions precisely similar to the *spotted* fever, which was so prevalent at that time in the London Hospitals.

"In the spring of 1822 puerperal fever existed in the Lying-in Hospital in two very different and well-marked forms, at an interval of about six weeks between the last case of the first epidemic and the first case of the second. The early cases were of an active inflammatory character; the peritoneal covering of the uterus and intestines was chiefly affected; the albuminous and serous effusions in the fatal cases showed a sthenic state of the system; that is, the serum was clear, the coagulable lymph firm and white; the patients bore blood-letting and other active treatment to a great extent fairly, and with much advantage; the blood drawn was strongly cupped and highly buffed, and the fatal cases were few. Six weeks afterwards a very different epidemic was found to exist. The same remedies which had been so beneficial a few weeks before were naturally at first tried, but their bad success confirmed the sagacious remark of Gooch, that 'the effects of remedies form not only an essential but the most important part of the history.' (Gooch *on Peritoneal Fevers*, p. 35.) The fever was attended with marked oppression and debility; the local pain was comparatively slight; the pulse was extremely rapid from the first, with no force, and easily compressible; in many of the cases purulent deposits took place in the joints and in the calves of the legs, and in one case there was destructive inflammation of the eye. On dissection, a quantity of fetid, dark, turbid serum, with loose and soft shreds of dirty lymph, was found in the peritoneal cavity, with a large collection of highly offensive gas. In some the substance of the uterus and ovaries was infiltrated with pus, especially in those cases where there had been purulent deposits in the limbs. It was shortly after these cases occurred, that attention was directed by Dr. Marshall Hall to some similar cases of purulent deposit and destruction of the eye after parturition, (*Med. Chir. Trans.*, vol. xiii. part 1,) and which were the first series of cases published in this country of that nature, although some isolated cases had been previously noticed, which had been looked on as accidental complications. In these two epidemics, so striking a variety of character could not fail to attract attention, and we shall shortly have occasion to notice others; and yet Dr. Armstrong quotes with approbation the following sentence from Dr. Hulme :—'The operations of nature upon the human frame in this disease are the same in Britain as in Greece, and continue the same at this day as they were above two thousand years ago. This is likewise a clear proof of the IMMUTABILITY of puerperal fever.' (Armstrong, p. 63.) In our opinion the puerperal fevers vary, as other fevers do, according to the season, local symptoms, the effects of remedies, and in the organs affected. We shall not trace the history of the numerous epidemics which have been recorded; but referring those who are desirous of acquiring such information to the more extended publications on puerperal fever, proceed to give a plain and practical account of those forms of the disease which are met with in hospitals and private practice, cautioning young practitioners to reflect, that as these epidemics have already so much varied, new varieties may again be found; and that it is advisable, especially for those who have the charge of lying-in hospitals, to watch closely and anxiously the first cases of the season, both as to the symptoms, and as to the effects of remedies, before deciding on the character of the disease. A

minute and searching investigation into the morbid appearances of the fatal cases is no less necessary, for there is fair reason to suppose, since the publication of the cases of inflammation of the veins, absorbents, and muscular structure of the uterus, by Dr. Robert Lee and others, that formerly those peculiar affections may have existed and yet escaped observation. It is to Dr. Robert Lee that the profession in this country is principally indebted for a much more extensive and complete investigation into the morbid changes produced in the course of this disease. But though agreeing with him to a certain point, and not doubting that in many, perhaps most of the cases formerly known as the low or malignant form of puerperal fever, the fatal symptoms have arisen from disorganizing inflammation of the deeper seated tissues of the uterus or its appendages, or from phlebitis, these changes having been overlooked in dissection; yet Dr. Lee has in our judgment made a material omission, and one very common at the present day, in passing over the influence of the nervous system in these cases; the vascular system is held to be all in all, everything is inflammation, and the powerful effect of altered nervous energy in the production of disease is lost sight of."

Consistently with these views, the author recommends a treatment adapted to the circumstances of each individual case, having regard to the character of the prevailing epidemic, and the effects of the treatment employed. General and local bleeding, fomentations, with mercury and opium, if there be unequivocal symptoms of inflammation with vigour, with an avoidance of general bleeding, and a greater reliance on local and tranquillizing means in adynamic cases.

Thirty years experience in the treatment of puerperal diseases, in hospital and private practice, has satisfied us of the justness of these views. The notion of Armstrong and others of the "*immutability of puerperal fever," has done incalculable mischief.*

As we have said, nearly one half of the matter in the present volume is from the pen of Dr. Clymer. These contributions are scattered throughout the work, and are either thrown into the body of the text or in foot notes, and always in brackets to distinguish them from the original.

On the subject of *Typhoid Fever*, Dr. C. has given us, as has been stated, a separate chapter of more than thirty pages, in which he has carefully embodied the views of American authorities.

"Typhoid Fever, [he observes,] is an acute affection, whose anatomical character is an enlargement and special alteration of the intestinal follicles, accompanied by increase of volume, injection, softening, and occasionally suppuration of the corresponding mesenteric gland."

The inference from this language would seem to be that Dr. C. regards this form of fever to be merely symptomatic of the intestinal lesions,—a position maintained by some modern writers, but as we believe without foundation,—such, however, as we glean from other passages, is not his opinion, but on the contrary, he looks upon it as "*an essential fever.*"

The assumption by our author, after Littré and others, that the disorder of the glands of Brunner and Peyer, constitutes the "*anatomical character*," may be well enough, as indicating that such a condition *generally* occurs; but this should not cause us to lose sight of the important fact noticed by Andral and other good observers, that it is sometimes absent, while softening of the liver, parietes of the heart, inflammation and ulceration of the mucous membrane of the bowels, all occur so frequently as to almost justify us in including them as anatomically characteristic of the disease; still less should we forget that these lesions occur in the progress of the fever, and are therefore concomitant or consequential, and not causative. And yet, in treating of the "identity of typhus and typhoid fever," Dr. C. assumes the existence of a diseased condition of the glands of Peyer and Brunner as the "distinctive or pathognomonic trait," as being "necessary to constitute the disease;" which, if typhoid fever may exist and run on to a fatal termination without any such manifestation, as Andral has reported, and as we have ourselves seen, then such a condition of these bodies is not "necessary to constitute the disease."

These expressions, taken in connection with the fact that the follicular derangements are not mentioned among the "secondary lesions," are certainly calculated at first view to cause the belief that Dr. C. looks upon typhoid fever as symptomatic rather than idiopathic or essential in its character.

Recent analyses of the blood show an altered condition of this fluid to be quite as constant a condition in typhoid fever as the follicular lesions to which so primary a regard has been had.

In relation to the "identity of typhus and typhoid fever," Dr.

C. is at issue with some of his countrymen as well as Europeans. His concluding remarks on this subject are as follows:

"We would inquire whether the group of symptoms known as typhoid fever is perfectly identical at all times and at all places; and further, if the essential characters of typhus are so constant or defined, that it can be taken as a fixed term of comparison? Do not epidemics of typhoid fever differ in their prominent phenomena, constantly from sporadic cases, as well as from each other? Are there not a number of accessory circumstances, so subtile as to escape observation, perpetually modifying the influence of causes on the organism? General conclusions should not be drawn from limited observations, or from witnessing any disease so generally prevalent as the one under consideration, in a single district, or even country, and during a limited period of time. And we cannot avoid thinking that those who regard these two forms of fever as distinct and separate *species*, are premature in the expression of an opinion upon a subject in which there are, as yet, wanting many essential elements to warrant a positive judgment."

Giving up the doctrine of a difference in identity of typhoid and typhus fever, is surrendering the point that follicular alteration constitutes the "anatomical character," or "distinctive pathognomonic trait" of the former, as no one contends for such anatomical character or "distinctive pathognomonic trait" in the latter of these diseases.

Dr. C. has evidently been most cautious in his efforts to avoid committing himself on the identity of *typhoid* and *typhus* fever, and in doing so, it appears to us, has sometimes expressed himself ambiguously.

We have not the time at present, nor is it our desire to enter into a full discussion of the mooted points contained in the present volume, and especially the subjects to which we have more particularly referred, and we here close our remarks with the expression of our thanks to Dr. Clymer for the large amount of useful matter he has added to these valuable dissertations.

*Five Dissertations on Fever.* By George Fordyce, M. D., F. R. S., Fellow of the Royal College of Physicians, Senior Physician to St. Thomas's Hospital, etc. *Second American Edition, with an Introduction.* 8vo. pp. 403. Ed. Barrington and Geo. D. Haswell. Philadelphia, 1846.

Dr. Fordyce, although not by any means an elegant writer, was an industrious contributor to medical literature. The most celebrated of his productions are the dissertations before us, and on them chiefly must rest his claims to present and future fame. Since they were composed, however, a better acquaintance with minute anatomy and the modifications of structure proceeding from disease, as well as the light shed on pathology by an improved knowledge of animal chemistry, have materially changed the views generally entertained of the etiology and essential characters of the different forms of fever, and, as a consequence, the treatment also. A greater reliance on the sanative powers of the system, and less on the use of perturbating remedies, may be said to characterize the modern practice as conducted by well instructed therapeutists. In this respect, our author may be said to have been in advance of his contemporaries. His antimonial treatment may be adduced as evidence of the correctness of this remark. The administration of the article in very minute quantities with a view to its alterative effect, instead of decided doses, as practised by others, was an improvement in practice, although proceeding from a questionable hypothesis. While other disturbing agencies were thus avoided, the remedy employed was in amount scarcely sufficient to interfere essentially with the natural course of the recuperative powers.

From these remarks it will be perceived, that although we accord to Fordyce much credit for his observations on the important class of diseases which constitute the subjects of these dissertations, we cannot receive them as entirely up with the present advanced state of our knowledge, or in any wise superseding the more recent works of authors on the same topics; still, the publishers have done an acceptable service to the profession in republishing them, and so enabling all who wish to store their libraries with standard authorities to possess themselves of a well printed copy, which is also enriched with an introductory chapter by the American editor.

---

*A Dictionary of Medical Science, containing a concise account of the various subjects and terms; with the French and other synonymes; Notices of Climate, and of Celebrated Mineral Waters; Formulæ for various Officinal and Empyrical Preparations, etc.* By Robley Dunglison, M. D., Professor of Institutes of Medicine, etc., in Jefferson Medical College of Philadelphia. *Sixth Edition. Revised and greatly enlarged.* Royal 8vo. pp. 808. Lea & Blanchard. Philadelphia: 1846.

The Dictionary of Professor Dunglison might, without any great stretch of propriety, be called a Cyclopædia of Medical Science, so comprehensive is it in its scope and so minute in its details. In this respect it possesses a great advantage over its contemporary publications of the kind. Whatever one may expect to meet with in a dictionary of terms, he is pretty sure to find in its ample pages, besides a vast deal of matter not commonly embraced in medical lexicons, and not readily found in other publications. Another great advantage is, that, from its rapid sale, and not being stereotyped, the author is enabled in the successive editions not only to supply any deficiencies, but to keep up with the rapid march of medicine and the collateral sciences, by embracing all the new terms rendered necessary or popular by the constant improvements going on. The same care and industry in posting displayed by the author in all his other publications, are evinced in this; of which some idea may be formed from the fact, that although but two years have elapsed since the last or fifth edition of the work came from the press, yet the present "comprises nearly *two thousand five hundred* subjects and terms not contained in the last." Many of these, as the author remarks, have been introduced into medical terminology in consequence of the progress of the science; and others had escaped him in previous editions."

*Address to the Graduates of the Philadelphia College of Pharmacy. Delivered April 15th, 1846.* By JOSEPH CARSON, M. D., Professor of Materia Medica and Pharmacy. *Published by request of the Board of Trustees.*

Philadelphia, we believe, is the only place on the continent of America where a College exists for the special cultivation and teaching of pharmacy, and it is to this circumstance that her druggists and chemists owe their acknowledged superiority in the science and practice of their professions. The address of Professor Carson inculcates a proper feeling and right appreciation of the value of their pursuits on the part of the young pharmaciens to whom he addressed himself.

The Trustees did wisely to request the publication of the address, which, within the brief space of fourteen pages, contains many useful hints and much apt counsel that should be constantly before the minds of their junior brethren.

---

*A popular Treatise on the Diseases of the Teeth, etc., etc.* By ROBERT ARTHUR, D. D. S., member of the American Society of Dental Surgeons, etc., etc. 12mo. pp. 187. Lindsay & Blakiston, Philadelphia: 1846.

In a former number we noticed the New York edition, published in 1845, of this popular treatise on Dentistry. It is a good manual, and comprises all that is really necessary for the general reader and practitioner of Medicine to know on the subject, and being written in a plain and perspicuous style, without the unnecessary use of technicalities, is adapted to readers of every class. The Philadelphia copy is in a handsome cloth cover, and presents quite a neat appearance.

---

*The Young Stethoscopist, or the Student's Aid to Auscultation.* By HENRY L. BOWDITCH, M. D. 12mo. pp. 278. New York—J. & H. G. Langley: Boston—William D. Ticknor & Co. 1846.

This is an exceedingly clever little work—clear, concise, and to the purpose. We know of no work on the subject better adapted to the wants of the student who is just beginning the study of auscultation.

